# Leading Change from Within: Student-Led Reforms to Advance Anti-Racism within Medical Education

**DOI:** 10.5334/pme.1076

**Published:** 2023-10-16

**Authors:** Tyler S. Warnock, Priatharsini Sivananthajothy, Whitney Ereyi-Osas, Pamela Roach

**Affiliations:** 1 University of British Columbia, Vancouver, Canada; 2 McMaster University, Hamilton, Canada; 3 Departments of Family Medicine and Community Health Sciences, University of Calgary, Calgary, Alberta, Canada; 4 Indigenous health education in the Office of Indigenous, Local and Global Health for the Cumming School of Medicine, Calgary, Alberta, Canada

## Abstract

Racism, physician biases against Indigenous, Black, and racialized people, and the resultant poor health outcomes have been the subject of many institutional position statements and calls to action. Across Canada, undergraduate medical education programs have recognized the importance of addressing racism, but material changes to curriculum and learning environments to incorporate anti-racist lenses have yet to be actualized. To bridge a gap seen within the curriculum, the authors of this manuscript led the co-development, organization, and implementation of a student-led anti-racism initiative at the University of Calgary’s Cumming School of Medicine. The initiative consisted of a class-wide anti-racism training session and a strategic review of student governance policies, including elections and decision-making processes through an anti-racist lens to advance equity within student learning environments. Anti-racism praxis was embedded within the co-creation of the anti-racism training by incorporating cultural safety and ethical engagement principles along with paid consultations with racialized students and faculty to identify pertinent topics and inform training priorities. Through this initiative, the authors offer an approach for the larger medical community to consider in their own local efforts to advance anti-racism advocacy and curricular change. This initiative highlighted the unique role of students in disrupting the status quo and modeling an anti-racist lens in their actions and self-governance.

## Background & Need for Innovation

In 2020, the killings of George Floyd, Breonna Taylor, and Joyce Echaquan led to an outpour of calls for action to dismantle systemic racism within medicine in Canada [1,2]. While contemporary literature has long documented systemic racism faced by Indigenous, Black and racialized people in healthcare settings, these events along with the resurgence of the Black Lives Matter movement were critical in forcing the medical profession to reflect on its complicity in maintaining the status quo [3,4,5]. In Canada, addressing anti-Indigenous and anti-Black racism requires significant attention due to the respective histories and experiences of colonization, intergenerational trauma, and enslavement leading to the resultant and continued marginalization of Indigenous and Black communities [1,6].

Indigenous people continue to encounter anti-Indigenous racism and discrimination in emergency departments in Canada, including “stereotyping, unacceptable personal interactions and poor quality of care” and unsafe care environments [5,7]. Moreover, Indigenous healthcare workers also frequently encounter racism from trainees, colleagues, and individuals in positions of higher authority [5,7].

Within the healthcare system, both anti-Indigenous and anti-Black racism often manifests in the forms of stereotyping and provider bias [8,9]. Consequently, it is critical for medical education programs to enable medical students to identify and address stereotyping and biases, build capacity for anti-racism practice, and become equipped with techniques to dismantle racist structures [1,3,5,9]. As future physicians, medical students are also bound to professional competency standards, such as the CanMEDS framework [10]. There is a wide call for anti-racism to underpin current CanMEDS competencies and the upcoming review provides a crucial opportunity for systemic change [11].

Developing anti-racism competencies within medical education will require institutions to reimagine the teaching and training of medical students to engage in anti-racism, anti-oppression, and uncovering of (un)intentional interpersonal racism [1,3]. Self-reflexivity and self-awareness of biases and stereotypes must be situated within socio-cultural, political, and historical contexts to highlight unequal structures of power that are maintained within healthcare institutions, ideologies, and cultural norms [4,12].

Historically, medical education has focused on cultural sensitivity or cultural competence training. Cultural competency, or the ability to work effectively within a cross-cultural context, is an important step towards creating anti-racist physicians, although it does not encompass this in its entirety [13]. While beneficial, cultural competency training has been critiqued for teaching only the understanding of others’ cultures, while lacking the strategies to recognize and dismantle power dynamics that perpetuate oppression and racism [14]. Thus, anti-racist curricula moves beyond working within cross-cultural contexts, by incorporating an understanding of the historical and present structures in society which perpetuate racism against racialized people and requiring a consistent effort to dismantle them on both an interpersonal and institutional level [14].

While anti-racism reform has historically been missing within medical education, a number of prominent external directives continue to call for reform, including the Truth and Reconciliation Commission, National Inquiry into Missing and Murdered Indigenous Women and Girls, the Indigenous Physicians Association of Canada, and Black Physicians of Canada. These national bodies have long advocated for healthcare institutions to address anti-Indigenous and anti-Black racism in medical education [1,4,6,15]. While institutions increasingly recognize their role in dismantling racism through changes in medical education, there has been little success in incorporating mandatory anti-racism education within undergraduate medical education programs. Multiple barriers exist to implementing anti-racism reforms, including overcrowded curriculums, lack of faculty expertise, limited resources, or limited decision-making power [16]. Additional barriers to student-led reforms must also be considered, such as hierarchical power dynamics and risks to training when engaging with faculty leaders. Despite the competing priorities within the medical curricula, anti-racism must be prioritized if medical education institutions are to genuinely respond to the broader calls to action and create safe environments for Indigenous, Black, and racialized patients and healthcare providers, alike. While institutions have been quick to publicize their support for anti-racism reforms, operationalizing these changes with the incorporation of anti-racism concepts and upholding safe learning environments for racialized students has yet to actualize, thus questioning if medical schools are truly able to uphold their social accountability mandates [17]. This paper aims to discuss one avenue that medical students have pursued to further advocate for the inclusion of anti-racism training into their learning environments.

## Goal of Innovation

Recognizing the shortcomings that Canadian medical education has in addressing anti-Indigenous and anti-Black racism, a team of medical students from the University of Calgary introduced an initiative to address curricular gaps in anti-racism teaching and to reimagine student governance through an anti-racist lens [1,4,18]. This led to the creation of the Students Organized Against Racism (SOAR) initiative, which was implemented between January and November 2021, through the Calgary Medical Students’ Association (CMSA). The goal of the SOAR initiative was to provide training sessions to medical students to introduce the concept of anti-racist frameworks and offer specific and relevant strategies to addressing racism when encountered on interpersonal and systemic levels within the healthcare environment. Secondly, SOAR was also aimed towards reforming student governance structures with an anti-racist framework to further the CMSA’s mandate to support and advocate for safer training and learning environments for medical students.

As a student-run initiative, SOAR served as an opportunity to showcase the ability of medical students to rapidly identify gaps within their own learning environment and develop solutions that were relevant and responsive. This process allowed for us to openly critique the absence of anti-racism teaching within the curriculum, while also encouraging the development of professional resistance [19]. Everyday experiences of microaggressions, racism, and the dominant focus on biomedical education in our curriculum inspired our pursuit for explicit teaching on anti-racism. When requests for protected curriculum time were rejected, we began this initiative balancing our roles as students, who faced potential professional repercussions, but also as members of an imperfect education system, who were driven by a social and moral imperative to raise our collective understandings of anti-racism [19].

As medical students ourselves (TSW, PS, WEO), our own positionality informed the design and approach to this initiative. TSW is a Japanese-Canadian male. PS is a Thamil-Canadian woman whose family fled from the Thamil genocide in Sri Lanka. WEO is a second-generation Nigerian-Canadian woman. Our team was also supported by our principal investigator, PR, who is a Michif and British woman, and a member of the Métis Nation of Alberta. As authors of color, participating in this work holds a deeper meaning due to our own personal and communal histories with racism.

## Steps Taken for Development and Implementation of Innovation

There is a paucity of literature in Canada on what anti-racism training in medical education entails, with even fewer anecdotal instances of how students can support this work [18]. With calls for medical schools to integrate structural competencies and skill-building to address structural oppression current students are in a grey area; incoming reforms are still in progress, yet students are continuing to matriculate through programs without formal teaching on anti-racist practice. Thus, we developed a rapid intervention to address these shortcomings. We recognize that the SOAR initiative, and similar student-run initiatives, are one of many tools that can be utilized to advance anti-racism within medical education. For student leaders in other institutions, the basic components of the SOAR initiative, namely anti-racism training as well as policy reform, are tangible changes that can be adapted and implemented into different contexts to address local needs.

**Figure 1:**
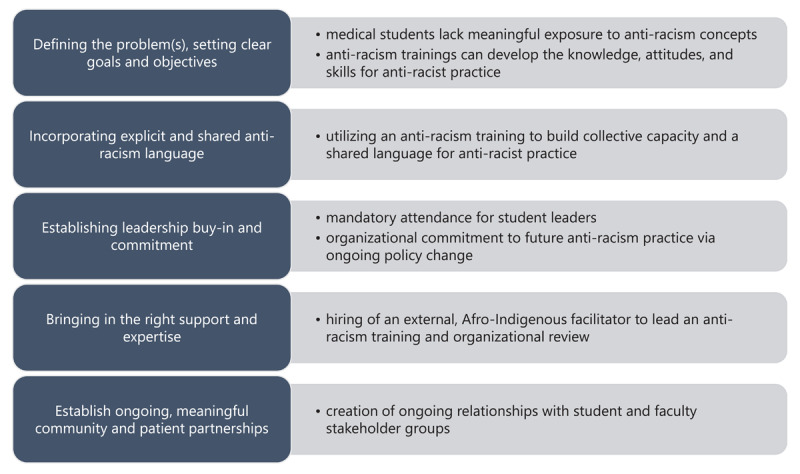
Process for developing and implementing the SOAR initiative.

**Figure 2:**
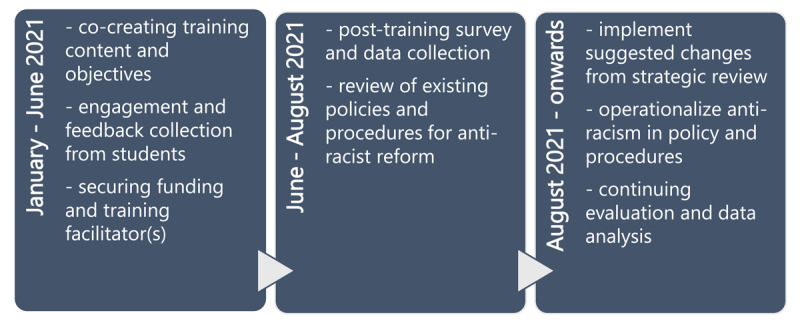
Timeline of the medical student-led SOAR program at the University of Calgary.

Guided by Hassen’s principles for anti-racism interventions in healthcare, we outline the process for implementing the SOAR initiative in Figure 1 [18]. While the process is laid out in a linear fashion in Figure 1, many of these steps are iterative and simultaneous as we worked through each of the principles during co-creation efforts. These processes played out over the course of eleven months through three phases, which are outlined in Figure 2.

We incorporated Bennett and Keating’s key factors for effective race equity training when co-creating this initiative [20]. These included considerations for how this training fit within broader organization policies on anti-racism, the facilitator’s qualifications, ensuring there was no cost to attend, and the commitment of participants in the training itself [20]. Although we originally envisioned this initiative as a one-time anti-racism training for medical students, we pivoted to include a program evaluation and organizational review to create a robust anti-racist policy alignment.

Working in line with the principles of bringing in the correct expertise as outlined by Hassen, we opted to work with a local Afro-Indigenous anti-racism facilitator to co-create the anti-racism training session and facilitate the strategic review of the CMSA. Recognizing our own limitations as medical learners, we chose to secure an external facilitator with an extensive background in anti-racism training to lead both the full-day anti-racism training and the strategic review. The objectives of the training were to improve student capacity to identify individual and institutional forms of systemic racism, explore mechanisms which advance systemic racism and employ anti-racist techniques and skills to address interpersonal and institutional forms of racism. Objectives were purposefully broad given the short nature of the training and to further allow participants to adapt learnings to their own individual anti-racism journey. The training was delivered virtually, due to the nature of the COVID-19 pandemic, with a combination of didactic teaching, group discussion, and individual reflection time.

To identify and ensure that the anti-racism training addressed current gaps in the curriculum and to establish ongoing, meaningful relationships with stakeholders, current medical students and faculty advocates of colour were recruited as part of the co-creation of the training sessions. Initial engagement sessions held with six students, including two Indigenous, two Black, and two racialized students, aided in identifying initial local barriers to actualizing anti-racist practice in medicine, along with other pertinent topics to include in the training that were missing from our curriculum. We also engaged with a Black clinical faculty member who provided feedback and suggestions for drawing further connections between anti-racism and clinical practice. All students and faculty were provided an honorarium for their time, expertise, and participation. The feedback from these engagement sessions were compiled and shared with the external facilitator to ensure the training content would be aligned with student and faculty identified priorities.

In order to establish leadership buy-in and commitment, we advocated to faculty leaders at our medical school for the anti-racism training to be a component of the curriculum; however, we were unsuccessful in securing a mandatory space within our program. Thus, the training was delivered as an optional, extra-curricular activity for current medical students. The CMSA exemplified their buy-in by mandating the training as required for students in leadership positions within the organization. Ideally, anti-racism training should be a required component of medical curricula, therefore ensuring all learners attend, regardless of interest, and would have exposed this content to a wider audience. Unfortunately, given administrative barriers within our training environment, this negatively impacted the ability for this training to reach student participants who may have benefitted the most. Since effective anti-racism training often requires mandatory participation, this is an area for future advocacy in subsequent training sessions [18].

## Outcomes of Innovation

In total, 50 students engaged in the full-day, seven-hour long online training session.

Following the training, twenty-four student leaders undertook a strategic review of the CMSA. The objective of this review was to incorporate anti-racist praxis into student governance practices so that anti-racism not only underscored interactions with the student body, but also in future advocacy efforts with faculty leaders. A virtual workshop with the external facilitator was held where CMSA student leaders, students-at-large, and faculty contributed to a dialogue on the paths forward for transforming CMSA structures, policies, and practices to become anti-racist.

The strategic review generated further discussion and commitments from student leaders to reform CMSA policy and procedures. The key outcomes from these discussions included changes to nomination processes for student representation on medical school admissions committees, new accountability mechanisms and communications strategies between student leaders and the broader student body, and a longitudinal commitment for further anti-racism training. A commitment to applying anti-racist and anti-colonial lenses in all policies and programs was also adopted. Processes to incorporate learnings from these sessions are ongoing, with the aim that the CMSA can help support anti-racism initiatives on a broader student level.

## Critical Reflection

While the SOAR initiative generated positive outcomes from its first year, this project and the efforts taken by students to advance anti-racism changes within medical education will never be truly complete. As this project was led by senior medical students, ensuring that new medical students and faculty stakeholders can continue this initiative for future cohorts is pertinent for long-term success. Institutional support which allows for incorporation of anti-racism training as a mandatory element of the curriculum is also important for long-term success and uptake by the student body. Forthcoming program evaluations from the SOAR initiative are also underway to ensure that the student perspective and experience can be documented and shared within the literature.

Student initiatives allow for rapid interventions to address gaps in medical education due to the ease of decision-making by a small leadership team, rapid incorporation of feedback from students and flexibility with funding. Modelling anti-racist praxis during the planning and co-creation of the SOAR initiative was an important priority identified, as our process in organizing this initiative needed to uphold cultural safety and ethical engagement. Having meaningful engagement, with paid honorariums, offered an opportunity to collect valuable feedback and acknowledge the time and expertise of racialized students and faculty.

While student-led initiatives, such as the one described in this article, can quickly bridge gaps, we recognize that the overall impact of such an initiative is limited. One of the challenges was having limited leadership support from the medical school itself. While lack of space in the curriculum was one barrier, ultimately, furthering anti-racism training in our curriculum is a task that will require institutional leadership buy-in and support. As a student-led initiative, we are also cognizant of the significant labor associated with curricular reform, especially the psychological burden of racialized students leading anti-racist reforms. Ideally, anti-racism reforms in curricula should not require unpaid student labor to materialize. However, continued advocacy for long-term curricular changes and ensuring accountability of medical school leadership often falls on the shoulders of student leaders [17]. Reorienting medical education to better address structural causes of oppression and racism within healthcare will be a significant, but necessary undertaking that must be driven by faculty and institutional leaders in their mandate to keep medical programs socially accountable.
